# Common Earache

**Published:** 1880-11-20

**Authors:** Thomas F. Houston

**Affiliations:** Atlanta, Ga.


### Southern Medical Record.

EDITORS:

T. S. POWELL, M.D. W. T. GOLDSMITH, M.D. R. C. WORD, M.D.

*R. C. WORD, M.D., Managing Editor.*

☞All Communications and Letters on Business connected with the RECORD must be addressed to the Managing Editor.

VOL. X. ATLANTA, GA., NOVEMBER 20, 1880. NO. 11.

### ORIGINAL AND SELECTED ARTICLES.

### COMMON EARACHE,
*Alias*
### ACUTE CATARRHAL INFLAMMATION OF THE MIDDLE EAR.

BY THOMAS F. HOUSTON, M.D., ATLANTA, GA.

**Symptoms.**—*Subjective.* 1. Pain in the depth of the ear.

2. Sense of fullness in the same part.
3. Noises in the ear.

*Objective.* 1. Vascular injection.

2. Bulging of the membrana tympani.
3. Impairment of the hearing.
4. Catarrh of the pharynx and eustachian tube.
5. Fever.

Patients with close powers of observation may notice that the throat is sore, and that the pharynx felt thickened before the pain commenced; also a sense of fullness in the ears. But generally the pain jumps to its full height at one bound. It will, if not arrested, pass over this stage, and result in acute suppuration of the middle ear. Some physicians seem to invite this state of affairs by such remarks as this: "It is a rising in the ear, put some poultices to it and draw it to a head."

The diagnosis of this disease is quite difficult in very young children; however, if we watch an infant closely we can narrow the pain down to the head, and then by pressure on the tragus, closely observing if the child winces, will enable us to decide if the affection is aural in character.

With certain easy-going practitioners "cutting a tooth" is the diagnosis made for many painful and obscure symptoms. Thus they temporise by cutting the gums, etc., until suppuration takes place, and the bungler has to repent allowing nature to make his diagnosis for him. The pouring of warm water into the ear will usually temporarily relieve an infantile earache, and in this way we have a means of diagnosis always at hand. At times, however, this will fail, and we have to depend upon objective symptoms. They are chiefly found in the membrana tympani. This is sometimes of a pinkish hue. Sometimes the periphery, and along the handle of the maleus, are the only parts showing vascular injection, the rest of the membrane being clear. Then again this redness is so intense that nothing can be seen save an evenly red surface, that has no perceptible vessels. If the ear has been troubled in this way before, the drum membrane will be rigid, thickened and opaque, and will show much more localized redness than intense vascular injection. Even when there is acute inflammation going on, the membrane may present only the appearance of a mirror that has been breathed upon, without an increased redness. Impairment of the hearing is by no means the rule. In the first stage of the disease—that is the stage of pain—it may be painfully acute, especially during cases of acute inflammation supervening on chronic aural catarrh.

Bulging of the membrane will probably occur after the first forty-eight hours; if the disease continue longer rupture of the membrane is likely to result. This intumescence of the membrane is in the posterior and inferior quadrant and in Shrapbnell membrane. In rare cases the protruding membrane may be observed to pulsate synchronously with the heart. The patient almost always has fever; sometimes the temperature is quite high, yet the average physician uses none of the antiphlogistic remedies he would employ were any other organ similarly affected.

*Causes.*—Of the almost numberless causes and influences that can produce this affection may be mentioned wet feet, drafts, ducking the head under water, constitutioual diseases such as small pox, scarlet fever and measles, in which the throat is attacked; also pneumonia and bronchitis, syphilitic affection, etc., also the nasal douche. This is, however, very rarely the case; I have found but three or four cases mentioned in investigating aural literature with regard to this subject.

*Treatment.*—The antiphlogistic treatment is beyond a doubt the proper one for this affection, viz: laxatives, local bloodletting and opium. Poultices are remedies often used, and while they relieve the pain are likely to increase the danger of rupture of the membrana tympani, especially if they are used many hours in succession. In some cases we are compelled to use them to allay the pain, and when we do they should be made small enough to enter the external auditory canal, with but little covering on the auricle.

Local bloodletting is the chief and first remedy, by leeches applied to the tragus, *and not of the mastoid process.* In the absence of leeches I would prefer water as hot as can be borne, applied continuously with an aural douche. If leeches cannot be obtained, and warm water does not afford relief, apply cups, wet or dry, to the auricle. When we see that rupture of the drum is imminent, puncture of the membrane is demanded, using a cataract needle or like instrument. If the mastoid process is red and hot, paint with iodine, and if this does not relieve it—and should the inflammation increase—make an incision down through the periostium; this, however, is rarely necesssary. Use gargles to the pharynx, either chlorate potash or iodine gtts. xx to water ℥j, and apply a cloth, wet with warm water, around the throat. Should suppuration and perforation take place, syringe out the ear carefully three or four times daily, and pour in a half teaspoonful of a solution of zinc, grains iij to water ℥j, turning the head on one side to prevent the fluid escaping. If this does not stop the discharge, apply with a camel's hair pencil, the ear being well illuminated, a solution of nitrate of silver 40 to 60 grains to the ounce, after each syringing.

Politzer's method and the eustachian catheter, should be daily used after the acute symptoms have subsided, whether perforation takes place or not. Sometimes the vapor from hot water, or the smoke from a pipe or cigar, will give relief to the sufferer.

Test the hearing accurately when the pain has subsided, to see if any impairment has occurred. With regard to general treatment, keep the patient in a warm room during the pain and fever; if necessary give a full dose of opium at bed time. Give nourishing diet and daily baths. Should the patient be suffering from any grave constitutional disease, the practitioner should not neglect the aural catarrh, as the local treatment will interfere but little with the constitutional remedies, and if it is neglected may do inestimable damage, and even destroy life itself, because should suppuration result, it may lead to caries, pyæmia, cerebral abscess, polypi and meningitis. A patient had better die under the disease than recover only to linger on in misery from the terrible consequences of this affection.

I claim nothing new, strange or original in the preparation of this paper, but I have been struck with the ignorance of many of our most gifted and best informed "general practitioners" of the nature and treatment of "common earache." They have but little time, amid the busy round of their professional ward, to devote to specialties, and that most neglected is the ear. If I have been able to give such a one an idea, a hint that will enable him to give ease to some sufferer, and save from impairment that almost chiefest boon, *good hearing*, my paper has accomplished its object, and I am thereby paid for any trouble and labor that I have expended in its preparation.

